# (μ-3,4-Diacetyl­hexa-2,4-diene-2,5-diol­ato-κ^4^ *O* ^2^,*O* ^3^:*O* ^4^,*O* ^5^)bis­[aqua(1,10-phen­an­thro­line-κ^2^ *N*,*N*′)copper(II)] bis­(tetra­fluorid­oborate) monohydrate

**DOI:** 10.1107/S1600536809006898

**Published:** 2009-03-06

**Authors:** Jorge A. Tovilla, Simón Hernández-Ortega, Jesús Valdés-Martínez

**Affiliations:** a Instituto de Química, Universidad Nacional Autónoma de México, Circuito Exterior s/n, Ciudad Universitaria, 04510 Coyoacán, México, DF, Mexico

## Abstract

In the title compound, [Cu_2_(C_10_H_12_O_4_)(C_12_H_8_N_2_)_2_(H_2_O)_2_](BF_4_)_2_·H_2_O, the two Cu atoms are each chelated by the acetyl­acetonate unit of the 3,4-diacetyl­hexa-2,4-diene-2,5-diolate (tae) ligand. The Cu atoms are square-pyramidally penta­coordinated, with one bidentate 1,10-phenanthroline (phen) and the tae ligand basal and one water mol­ecule apical. The pyridyl rings of the phen ligands participate in π–π [centroid–centroid distance = 3.894 (3) Å] and C—H ⋯ π inter­actions, generating layers which are inter­connected through O—H⋯O and O—H⋯F hydrogen bonds between the water mol­ecules and the tetra­fluorido­borate anions. The F atoms of both tetra­fluorido­borate anions are each disordered over two positions of equal occupancy.

## Related literature

For related Cu(II)–tae^2−^–diimine complexes, see: Shen *et al.* (1999*a* ▶,*b* ▶); Lim *et al.* (1994 ▶); Fukuda *et al.* (1994 ▶); Zhang *et al.* (1999 ▶). For other similar metal complexes, see: Zhang *et al.* (1998 ▶, 1999 ▶); Mei *et al.* (2006*a* ▶,*b* ▶).
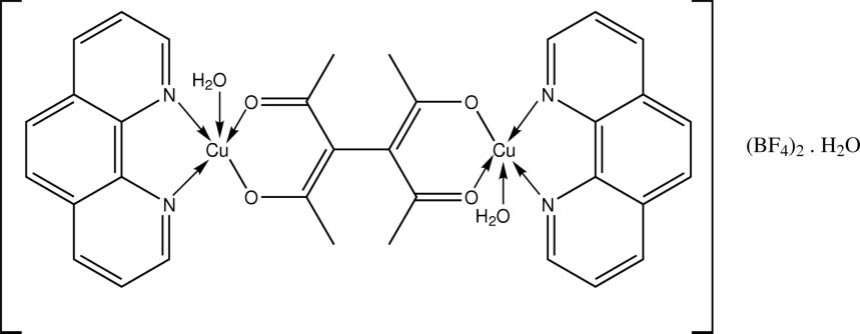


## Experimental

### 

#### Crystal data

[Cu_2_(C_10_H_12_O_4_)(C_12_H_8_N_2_)_2_(H_2_O)_2_](BF_4_)_2_·H_2_O
*M* *_r_* = 911.35
Triclinic, 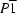

*a* = 11.5555 (9) Å
*b* = 12.0954 (9) Å
*c* = 15.4446 (12) Å
α = 67.654 (1)°
β = 78.890 (1)°
γ = 72.784 (1)°
*V* = 1899.2 (3) Å^3^
*Z* = 2
Mo *K*α radiation
μ = 1.21 mm^−1^
*T* = 298 K
0.16 × 0.08 × 0.04 mm

#### Data collection

Bruker SMART APEX CCD area-detector diffractometer
Absorption correction: multi-scan (*SADABS*; Sheldrick, 1996 ▶) *T*_min_ = 0.858, *T*_max_ = 0.949
15806 measured reflections
6928 independent reflections
3558 reflections with *I* > 2σ(*I*)
*R*_int_ = 0.059

#### Refinement

*R*[*F* ^2^ > 2σ(*F* ^2^)] = 0.055
*wR*(*F* ^2^) = 0.114
*S* = 0.84
6928 reflections
606 parameters
326 restraints
H atoms treated by a mixture of independent and constrained refinement
Δρ_max_ = 0.54 e Å^−3^
Δρ_min_ = −0.40 e Å^−3^

### 

Data collection: *SMART* (Bruker, 1999 ▶); cell refinement: *SAINT* (Bruker, 2006 ▶); data reduction: *SAINT*; program(s) used to solve structure: *SHELXS97* (Sheldrick, 2008 ▶); program(s) used to refine structure: *SHELXL97* (Sheldrick, 2008 ▶); molecular graphics: *X-SEED* (Barbour, 2001 ▶); software used to prepare material for publication: *PLATON* (Spek, 2009 ▶), *publCIF* (Westrip, 2009 ▶) and *enCIFer* (Allen *et al.*, 2004 ▶).

## Supplementary Material

Crystal structure: contains datablocks I, global. DOI: 10.1107/S1600536809006898/ng2549sup1.cif

Structure factors: contains datablocks I. DOI: 10.1107/S1600536809006898/ng2549Isup2.hkl

Additional supplementary materials:  crystallographic information; 3D view; checkCIF report

## Figures and Tables

**Table:** 

Cu1—O2	1.886 (3)
Cu1—O1	1.892 (3)
Cu1—N2	2.008 (4)
Cu1—N1	2.013 (4)
Cu1—O5	2.320 (4)
Cu2—O3	1.884 (3)
Cu2—O4	1.895 (3)
Cu2—N4	1.990 (4)
Cu2—N3	2.004 (4)
Cu2—O6	2.363 (5)

**Table:** 

O1—Cu1—N2	166.92 (15)
O2—Cu1—N1	171.40 (16)
O4—Cu2—N4	168.61 (17)
O3—Cu2—N3	172.57 (17)

**Table 2:** Hydrogen-bond geometry (Å, °)

*D*—H⋯*A*	*D*—H	H⋯*A*	*D*⋯*A*	*D*—H⋯*A*
O7—H7*B*⋯F1*A*^i^	0.845 (11)	1.91 (2)	2.737 (11)	166 (8)
O7—H7*A*⋯F6*A*	0.850 (11)	1.90 (3)	2.728 (9)	164 (8)
O7—H7*A*⋯F7	0.850 (11)	1.99 (4)	2.782 (8)	155 (8)
O6—H6*E*⋯F4^ii^	0.852 (11)	2.054 (18)	2.898 (9)	171 (7)
O6—H6*D*⋯F2*A*^iii^	0.852 (11)	2.080 (16)	2.930 (10)	175 (7)
O6—H6*D*⋯F2^iii^	0.852 (11)	1.90 (3)	2.694 (10)	155 (7)
O5—H5*E*⋯F3*A*	0.846 (11)	1.96 (2)	2.776 (8)	163 (6)
O5—H5*D*⋯O7^iv^	0.842 (11)	2.000 (15)	2.837 (7)	173 (6)
O7—H7*A*⋯F7	0.850 (11)	1.99 (4)	2.782 (8)	155 (8)
O7—H7*A*⋯F6*A*	0.850 (11)	1.90 (3)	2.728 (9)	164 (8)
C29—H29⋯*Cg*^ii^	0.93	2.75	3.522 (7)	141

Symmetry codes: (i) 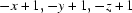; (ii) 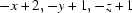; (iii) 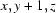; (iv) 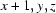. *Cg* is the centroid of the N1,C12,C11,C13,C21,C20 ring.

**Fig. 1.**
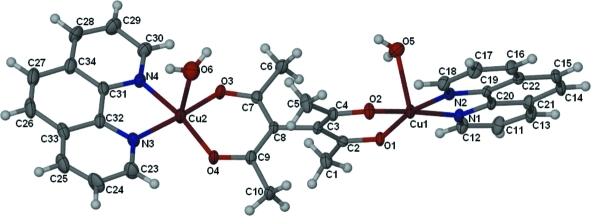
The molecular structure of the cation in (1), showing the atom- labeling scheme. Displacement ellipsoids are drawn at the 30% probability level and H atoms are shown as small spheres of arbitrary radius.

**Fig. 2.**
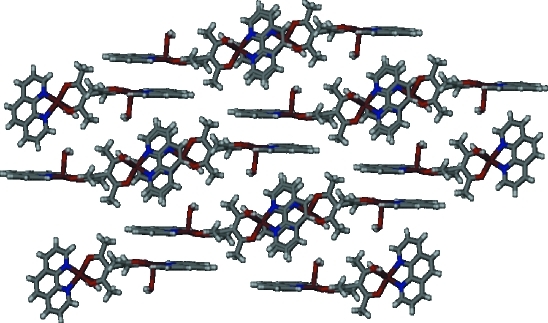
View of the molecular packing in (1).

## supplementary crystallographic information

### Comment

The asymmetric structure of 1 consists of one dinuclear [(phen)(H_2_O)Cu(tae)Cu(H_2_O)(phen)]^2+^ complex cation, two disordered BF_4_^-^ anions and a water molecule, where phen represents the 1,10-phenanthroline, and tae the 3,4-diacetylhexa-2,4-diene-2,5-diolate, see Figure 1. In the cation one Cu atom coordinates to each acac^-^ moiety of the tae^2-^. The Cu atoms are pentacoordinated with one bidentate phen and one water molecule in addition to the tae^2-^ ligand. The geometry around both Cu atoms is square pyramidal as indicated by the values of the τ parameter, 0.07 for both Cu1 and Cu2. The two acac moieties of the tae ligand deviate from the ideal geometry as indicated by the torsion angles C4—C3—C8—C7 and C2—C3—C8—C9, 99.9 (6) and 101.1 (6)°, respectively (ideal values 90°). The deviation of the ideal geometry is also reflected in the angle the central rings of the phen ligands C(14)—C(15)—C(22)—C(19)—C(20)—C(21) and C(26)—C(27)—C(34)—C(31)—C(32)—C(33) which has a value of 73.6 (3)°, ideal value 90°. The water molecule coordinated to Cu1 forms a hydrogen bond with the free water molecule, O(5) ···O(7) 2.835 (6) Å. In addition the three water molecules present interactions with the disordered BF_4_^-^ anions. Two of the py rings of each phen ligands coordinated to the Cu1 present π-π interactions between them: N(1)—C(12)—C(11)—C(13)—C(21)—C(20) and N(2)—C(18)—C(17)—C(16)—C(22)—C(19)[2 - *x*,-*y*,2 - *z*], have centroid···centroid distance, *Cg*···*Cg*, of 3.894 (3) Å. H-bonded dimers are formed through C—H ··· π interactions between one py ring of the phen coordinated to Cu2 and a phen coordinated to Cu1, C(29)—H(29)···N(1)—C(12)—C(11)—C(13)—C(21)—C(20) [2 - *x*,1 - *y*,1 - *z*] with a C···*Cg* = 3.522 (7) Å, Figure 2.

### Experimental

Copper(II) tetrafluoroborate hydrate (H_2_O-31.2%) (0.188 g, 0.69 mmol) was added to a freshly made mixture of 1,10-phenanthroline (0.124 g, 0.69 mmol) and tetraacetylethane (0.068 g, 0.34 mmol) in methanol (15 ml) to give a dark green-blue suspension. The reaction mixture was stirred for 3 hrs at 45°C in a water bath, firstly and then at room temperature overnight. The blue-green solid was recovered by filtration and it was air-dried. 12 mg of the product were suspended in acetone (1 ml) and water (*ca* 1 ml) was added in order to achieve complete dissolution. Crystals suitable for X-ray analysis were obtained after 2 weeks of slow evaporation.

### Refinement

Both BF_4_^-^ anions are disordered and were refined in two major contributors with s.o.f. 0.5. The H atoms on O atoms were located in the Fourier map and refined with U(iso)= 1.5*U*_eq_(H_2_O). H on C atoms were fixed geometrically and treated as riding with 0.96Å (methyl) and 0.93Å (aromatic) with *U*_iso_(H)= 1.2*U*_eq_(aromatic) or *U*_iso_(H) = 1.5*U*_eq_(methyl).

### Crystal data

**Table:**

[Cu_2_(C_10_H_12_O_4_)(C_12_H_8_N_2_)_2_(H_2_O)_2_](BF_4_)_2_·H_2_O	*Z* = 2
*M**_r_* = 911.35	*F*(000) = 924
Triclinic, *P*1	*D*_x_ = 1.594 Mg m^−^^3^
Hall symbol: -P 1	Mo *K*α radiation, λ = 0.71073 Å
*a* = 11.5555 (9) Å	Cell parameters from 3675 reflections
*b* = 12.0954 (9) Å	θ = 2.2–24.9°
*c* = 15.4446 (12) Å	µ = 1.21 mm^−^^1^
α = 67.654 (1)°	*T* = 298 K
β = 78.890 (1)°	Prism, green
γ = 72.784 (1)°	0.16 × 0.08 × 0.04 mm
*V* = 1899.2 (3) Å^3^	

### Data collection

**Table:**

Bruker SMART APEX CCD area-detector diffractometer	6928 independent reflections
Radiation source: fine-focus sealed tube	3558 reflections with *I* > 2σ(*I*)
graphite	*R*_int_ = 0.059
Detector resolution: 0.83 pixels mm^-1^	θ_max_ = 25.4°, θ_min_ = 1.9°
ω scans	*h* = −13→13
Absorption correction: multi-scan (*SADABS*; Sheldrick, 1996)	*k* = −14→14
*T*_min_ = 0.858, *T*_max_ = 0.949	*l* = −18→18
15806 measured reflections	

### Refinement

**Table:**

Refinement on *F*^2^	Primary atom site location: structure-invariant direct methods
Least-squares matrix: full	Secondary atom site location: difference Fourier map
*R*[*F*^2^ > 2σ(*F*^2^)] = 0.055	Hydrogen site location: inferred from neighbouring sites
*wR*(*F*^2^) = 0.114	H atoms treated by a mixture of independent and constrained refinement
*S* = 0.84	*w* = 1/[σ^2^(*F*_o_^2^) + (0.0366*P*)^2^] where *P* = (*F*_o_^2^ + 2*F*_c_^2^)/3
6928 reflections	(Δ/σ)_max_ = 0.001
606 parameters	Δρ_max_ = 0.54 e Å^−^^3^
326 restraints	Δρ_min_ = −0.40 e Å^−^^3^

### Special details

**Table:**

Geometry. All e.s.d.'s (except the e.s.d. in the dihedral angle between two l.s. planes) are estimated using the full covariance matrix. The cell e.s.d.'s are taken into account individually in the estimation of e.s.d.'s in distances, angles and torsion angles; correlations between e.s.d.'s in cell parameters are only used when they are defined by crystal symmetry. An approximate (isotropic) treatment of cell e.s.d.'s is used for estimating e.s.d.'s involving l.s. planes.
Refinement. Refinement of *F*^2^ against ALL reflections. The weighted *R*-factor *wR* and goodness of fit *S* are based on *F*^2^, conventional *R*-factors *R* are based on *F*, with *F* set to zero for negative *F*^2^. The threshold expression of *F*^2^ > σ(*F*^2^) is used only for calculating *R*-factors(gt) *etc*. and is not relevant to the choice of reflections for refinement. *R*-factors based on *F*^2^ are statistically about twice as large as those based on *F*, and *R*- factors based on ALL data will be even larger.

### Fractional atomic coordinates and isotropic or equivalent isotropic displacement parameters (Å^2^)

**Table:**

	*x*	*y*	*z*	*U*_iso_*/*U*_eq_	Occ. (<1)
Cu1	0.99590 (6)	0.18154 (5)	0.85485 (4)	0.0390 (2)	
Cu2	0.65472 (6)	0.87240 (6)	0.61594 (5)	0.0491 (2)	
O1	0.8415 (3)	0.2366 (3)	0.8079 (2)	0.0404 (9)	
O2	0.9820 (3)	0.3323 (3)	0.8705 (2)	0.0468 (10)	
O3	0.7359 (3)	0.7405 (3)	0.5728 (2)	0.0445 (10)	
O4	0.6140 (3)	0.7653 (3)	0.7374 (2)	0.0515 (10)	
O5	1.1038 (4)	0.2306 (4)	0.7078 (3)	0.0604 (11)	
H5D	1.115 (6)	0.301 (3)	0.693 (4)	0.091*	
H5E	1.049 (4)	0.241 (6)	0.674 (4)	0.091*	
O6	0.8423 (5)	0.8930 (4)	0.6395 (4)	0.0763 (13)	
H6D	0.835 (7)	0.968 (2)	0.632 (5)	0.114*	
H6E	0.904 (4)	0.849 (6)	0.618 (5)	0.114*	
O7	0.1659 (5)	0.4582 (5)	0.6594 (4)	0.0931 (15)	
H7A	0.194 (7)	0.456 (8)	0.707 (4)	0.140*	
H7B	0.184 (7)	0.521 (5)	0.618 (4)	0.140*	
N1	1.0160 (4)	0.0087 (3)	0.8585 (3)	0.0374 (11)	
N2	1.1408 (4)	0.0983 (4)	0.9301 (3)	0.0368 (11)	
N3	0.5590 (4)	1.0233 (4)	0.6458 (3)	0.0490 (12)	
N4	0.6619 (4)	0.9957 (4)	0.4867 (3)	0.0485 (12)	
C1	0.6712 (5)	0.3667 (5)	0.7313 (4)	0.0523 (16)	
H1A	0.6628	0.2878	0.7364	0.078*	
H1B	0.6768	0.4177	0.6662	0.078*	
H1C	0.6016	0.4055	0.7640	0.078*	
C2	0.7842 (4)	0.3498 (5)	0.7739 (3)	0.0348 (13)	
C3	0.8174 (5)	0.4515 (4)	0.7750 (3)	0.0348 (13)	
C4	0.9136 (5)	0.4354 (5)	0.8251 (4)	0.0396 (14)	
C5	0.9433 (5)	0.5441 (5)	0.8342 (4)	0.0616 (18)	
H5A	1.0111	0.5151	0.8712	0.092*	
H5B	0.8740	0.5870	0.8644	0.092*	
H5C	0.9635	0.5990	0.7729	0.092*	
C6	0.8686 (5)	0.5545 (5)	0.5733 (4)	0.0555 (17)	
H6A	0.8767	0.6032	0.5078	0.083*	
H6B	0.8397	0.4845	0.5806	0.083*	
H6C	0.9463	0.5268	0.5981	0.083*	
C7	0.7798 (5)	0.6312 (5)	0.6254 (4)	0.0406 (14)	
C8	0.7509 (4)	0.5814 (4)	0.7219 (4)	0.0356 (13)	
C9	0.6633 (5)	0.6503 (5)	0.7713 (4)	0.0425 (14)	
C10	0.6193 (5)	0.5913 (5)	0.8720 (3)	0.0569 (17)	
H10A	0.5594	0.6520	0.8933	0.085*	
H10B	0.6866	0.5578	0.9100	0.085*	
H10C	0.5838	0.5264	0.8772	0.085*	
C11	0.9811 (6)	−0.1545 (5)	0.8256 (4)	0.0592 (17)	
H11	0.9341	−0.1807	0.7984	0.071*	
C12	0.9525 (5)	−0.0316 (5)	0.8195 (4)	0.0507 (16)	
H12	0.8868	0.0236	0.7871	0.061*	
C13	1.0777 (6)	−0.2358 (5)	0.8714 (4)	0.0574 (17)	
H13	1.0963	−0.3180	0.8763	0.069*	
C14	1.2556 (6)	−0.2718 (5)	0.9568 (4)	0.0529 (16)	
H14	1.2809	−0.3543	0.9616	0.064*	
C15	1.3201 (5)	−0.2274 (5)	0.9931 (4)	0.0503 (16)	
H15	1.3892	−0.2794	1.0226	0.060*	
C16	1.3461 (5)	−0.0469 (5)	1.0248 (4)	0.0529 (16)	
H16	1.4143	−0.0943	1.0572	0.063*	
C17	1.3051 (5)	0.0741 (6)	1.0134 (4)	0.0594 (17)	
H17	1.3457	0.1106	1.0373	0.071*	
C18	1.2015 (5)	0.1449 (5)	0.9657 (4)	0.0490 (15)	
H18	1.1747	0.2280	0.9589	0.059*	
C19	1.1824 (5)	−0.0245 (4)	0.9411 (3)	0.0346 (13)	
C20	1.1134 (5)	−0.0718 (4)	0.9031 (3)	0.0348 (13)	
C21	1.1489 (5)	−0.1963 (5)	0.9111 (4)	0.0427 (14)	
C22	1.2846 (5)	−0.1003 (5)	0.9872 (4)	0.0425 (14)	
C23	0.5072 (6)	1.0331 (6)	0.7278 (5)	0.0693 (19)	
H23	0.5129	0.9621	0.7809	0.083*	
C24	0.4450 (6)	1.1451 (6)	0.7371 (5)	0.082 (2)	
H24	0.4086	1.1488	0.7955	0.098*	
C25	0.4372 (6)	1.2499 (6)	0.6606 (6)	0.077 (2)	
H25	0.3957	1.3256	0.6666	0.093*	
C26	0.4903 (6)	1.3473 (6)	0.4869 (6)	0.076 (2)	
H26	0.4489	1.4258	0.4873	0.092*	
C27	0.5463 (6)	1.3346 (6)	0.4065 (5)	0.072 (2)	
H27	0.5465	1.4043	0.3529	0.087*	
C28	0.6657 (6)	1.1947 (6)	0.3192 (5)	0.076 (2)	
H28	0.6674	1.2605	0.2631	0.091*	
C29	0.7220 (6)	1.0756 (6)	0.3223 (5)	0.080 (2)	
H29	0.7620	1.0605	0.2681	0.096*	
C30	0.7183 (5)	0.9776 (6)	0.4079 (4)	0.0602 (17)	
H30	0.7564	0.8976	0.4095	0.072*	
C31	0.6061 (5)	1.1119 (5)	0.4834 (4)	0.0475 (15)	
C32	0.5494 (5)	1.1274 (5)	0.5703 (4)	0.0458 (15)	
C33	0.4910 (5)	1.2440 (6)	0.5738 (5)	0.0595 (18)	
C34	0.6068 (6)	1.2147 (5)	0.4007 (5)	0.0570 (17)	
B1	0.8838 (5)	0.2213 (5)	0.5346 (4)	0.101 (2)	
F1	0.8098 (11)	0.3342 (7)	0.5272 (7)	0.138 (4)	0.50
F2	0.8189 (11)	0.1382 (9)	0.5615 (8)	0.145 (5)	0.50
F3	0.9664 (10)	0.1914 (12)	0.5938 (8)	0.165 (5)	0.50
F4	0.9393 (10)	0.2322 (10)	0.4465 (5)	0.152 (4)	0.50
F1A	0.8141 (11)	0.3279 (8)	0.4850 (7)	0.145 (5)	0.50
F2A	0.8125 (10)	0.1552 (9)	0.6042 (6)	0.109 (3)	0.50
F3A	0.9625 (9)	0.2425 (12)	0.5766 (8)	0.135 (4)	0.50
F4A	0.9420 (11)	0.1540 (10)	0.4814 (7)	0.180 (5)	0.50
B2	0.3294 (4)	0.4202 (4)	0.8750 (3)	0.0641 (16)	
F5	0.2337 (8)	0.4460 (12)	0.9365 (7)	0.091 (4)	0.50
F6	0.4163 (9)	0.3279 (7)	0.9241 (7)	0.100 (3)	0.50
F7	0.2957 (9)	0.3809 (7)	0.8148 (5)	0.098 (3)	0.50
F8	0.3687 (11)	0.5225 (8)	0.8272 (6)	0.123 (4)	0.50
F5A	0.2328 (8)	0.4162 (13)	0.9389 (8)	0.098 (4)	0.50
F6A	0.2915 (10)	0.4675 (10)	0.7880 (5)	0.149 (4)	0.50
F7A	0.3964 (9)	0.4925 (8)	0.8799 (7)	0.103 (3)	0.50
F8A	0.4019 (9)	0.3057 (6)	0.8903 (8)	0.102 (3)	0.50

### Atomic displacement parameters (Å^2^)

**Table:**

	*U*^11^	*U*^22^	*U*^33^	*U*^12^	*U*^13^	*U*^23^
Cu1	0.0431 (4)	0.0245 (4)	0.0444 (4)	−0.0034 (3)	−0.0095 (3)	−0.0074 (3)
Cu2	0.0615 (5)	0.0320 (4)	0.0425 (4)	−0.0019 (4)	−0.0037 (4)	−0.0083 (3)
O1	0.043 (2)	0.0210 (19)	0.052 (2)	−0.0037 (17)	−0.0079 (19)	−0.0084 (18)
O2	0.051 (2)	0.031 (2)	0.057 (2)	−0.0017 (18)	−0.019 (2)	−0.0131 (19)
O3	0.066 (3)	0.025 (2)	0.033 (2)	−0.0038 (19)	−0.0013 (19)	−0.0074 (17)
O4	0.066 (3)	0.026 (2)	0.040 (2)	0.0045 (19)	0.009 (2)	−0.0058 (18)
O5	0.064 (3)	0.057 (3)	0.056 (3)	−0.019 (3)	−0.003 (2)	−0.012 (3)
O6	0.083 (4)	0.069 (3)	0.081 (3)	−0.018 (3)	−0.017 (3)	−0.026 (3)
O7	0.110 (4)	0.082 (4)	0.092 (4)	−0.029 (3)	−0.034 (4)	−0.019 (3)
N1	0.045 (3)	0.025 (2)	0.037 (3)	−0.005 (2)	−0.008 (2)	−0.005 (2)
N2	0.039 (3)	0.030 (3)	0.038 (3)	−0.008 (2)	−0.004 (2)	−0.009 (2)
N3	0.054 (3)	0.039 (3)	0.046 (3)	0.000 (2)	0.002 (3)	−0.018 (3)
N4	0.053 (3)	0.037 (3)	0.044 (3)	−0.005 (2)	−0.003 (3)	−0.006 (2)
C1	0.047 (4)	0.038 (3)	0.070 (4)	−0.004 (3)	−0.020 (3)	−0.014 (3)
C2	0.031 (3)	0.037 (3)	0.034 (3)	−0.007 (3)	−0.003 (3)	−0.010 (3)
C3	0.041 (3)	0.025 (3)	0.034 (3)	−0.005 (3)	−0.007 (3)	−0.006 (2)
C4	0.048 (4)	0.025 (3)	0.044 (3)	−0.004 (3)	−0.004 (3)	−0.013 (3)
C5	0.064 (4)	0.039 (3)	0.088 (5)	0.000 (3)	−0.029 (4)	−0.028 (3)
C6	0.071 (4)	0.040 (3)	0.045 (4)	−0.009 (3)	0.006 (3)	−0.012 (3)
C7	0.040 (3)	0.031 (3)	0.048 (4)	−0.008 (3)	−0.002 (3)	−0.012 (3)
C8	0.037 (3)	0.025 (3)	0.037 (3)	−0.002 (2)	−0.003 (3)	−0.006 (3)
C9	0.047 (4)	0.034 (3)	0.039 (3)	−0.006 (3)	−0.010 (3)	−0.006 (3)
C10	0.061 (4)	0.040 (3)	0.043 (4)	0.002 (3)	0.004 (3)	−0.001 (3)
C11	0.085 (5)	0.039 (4)	0.061 (4)	−0.019 (4)	−0.021 (4)	−0.017 (3)
C12	0.063 (4)	0.036 (3)	0.050 (4)	−0.009 (3)	−0.021 (3)	−0.007 (3)
C13	0.084 (5)	0.027 (3)	0.055 (4)	−0.008 (3)	−0.011 (4)	−0.010 (3)
C14	0.067 (5)	0.028 (3)	0.048 (4)	0.008 (3)	−0.005 (3)	−0.011 (3)
C15	0.044 (4)	0.039 (4)	0.046 (4)	0.008 (3)	−0.006 (3)	−0.005 (3)
C16	0.043 (4)	0.049 (4)	0.052 (4)	−0.003 (3)	−0.016 (3)	−0.002 (3)
C17	0.060 (4)	0.060 (4)	0.063 (4)	−0.023 (4)	−0.022 (4)	−0.013 (4)
C18	0.051 (4)	0.040 (3)	0.054 (4)	−0.013 (3)	−0.009 (3)	−0.011 (3)
C19	0.039 (3)	0.027 (3)	0.030 (3)	−0.005 (3)	−0.002 (3)	−0.004 (3)
C20	0.044 (4)	0.023 (3)	0.029 (3)	−0.003 (3)	−0.004 (3)	−0.003 (2)
C21	0.058 (4)	0.029 (3)	0.038 (3)	−0.007 (3)	−0.004 (3)	−0.012 (3)
C22	0.037 (3)	0.038 (3)	0.040 (3)	−0.007 (3)	−0.004 (3)	−0.002 (3)
C23	0.085 (5)	0.060 (4)	0.054 (4)	−0.002 (4)	−0.001 (4)	−0.024 (4)
C24	0.089 (6)	0.072 (5)	0.083 (6)	0.012 (4)	−0.002 (5)	−0.053 (5)
C25	0.071 (5)	0.049 (4)	0.110 (6)	0.008 (4)	−0.013 (5)	−0.040 (5)
C26	0.071 (5)	0.033 (4)	0.118 (7)	0.002 (4)	−0.027 (5)	−0.020 (5)
C27	0.069 (5)	0.045 (4)	0.081 (5)	−0.004 (4)	−0.022 (4)	0.002 (4)
C28	0.085 (5)	0.057 (5)	0.053 (5)	−0.012 (4)	−0.007 (4)	0.012 (4)
C29	0.099 (6)	0.069 (5)	0.047 (4)	−0.016 (5)	0.002 (4)	−0.001 (4)
C30	0.069 (5)	0.053 (4)	0.049 (4)	−0.013 (3)	−0.003 (4)	−0.009 (4)
C31	0.038 (4)	0.032 (3)	0.063 (4)	−0.003 (3)	−0.011 (3)	−0.008 (3)
C32	0.046 (4)	0.031 (3)	0.055 (4)	−0.006 (3)	−0.009 (3)	−0.010 (3)
C33	0.050 (4)	0.044 (4)	0.082 (5)	0.007 (3)	−0.018 (4)	−0.026 (4)
C34	0.060 (4)	0.041 (4)	0.057 (4)	−0.007 (3)	−0.017 (4)	−0.002 (4)
B1	0.135 (6)	0.089 (4)	0.110 (5)	−0.047 (4)	−0.019 (4)	−0.051 (4)
F1	0.226 (9)	0.086 (5)	0.115 (9)	−0.035 (5)	−0.001 (7)	−0.059 (6)
F2	0.162 (8)	0.095 (6)	0.222 (11)	−0.062 (6)	−0.044 (8)	−0.068 (7)
F3	0.184 (8)	0.193 (11)	0.126 (7)	−0.122 (7)	−0.059 (6)	0.014 (8)
F4	0.147 (8)	0.162 (10)	0.122 (6)	0.018 (7)	−0.011 (5)	−0.064 (7)
F1A	0.203 (9)	0.121 (6)	0.101 (9)	−0.027 (6)	−0.052 (7)	−0.018 (5)
F2A	0.136 (7)	0.086 (6)	0.138 (7)	−0.059 (5)	−0.006 (5)	−0.054 (5)
F3A	0.173 (8)	0.131 (9)	0.125 (8)	−0.098 (6)	−0.050 (6)	−0.008 (6)
F4A	0.206 (10)	0.175 (10)	0.196 (10)	−0.052 (8)	0.043 (8)	−0.130 (8)
B2	0.077 (4)	0.059 (4)	0.067 (4)	−0.019 (3)	−0.012 (3)	−0.029 (3)
F5	0.087 (6)	0.099 (8)	0.100 (6)	−0.014 (5)	0.005 (4)	−0.062 (5)
F6	0.084 (5)	0.096 (6)	0.116 (8)	0.004 (5)	−0.046 (5)	−0.035 (5)
F7	0.139 (7)	0.092 (6)	0.084 (6)	−0.009 (5)	−0.039 (5)	−0.052 (5)
F8	0.173 (8)	0.087 (5)	0.102 (8)	−0.066 (5)	0.002 (6)	−0.008 (5)
F5A	0.083 (6)	0.094 (7)	0.135 (6)	−0.034 (5)	0.024 (5)	−0.065 (6)
F6A	0.185 (8)	0.168 (9)	0.091 (5)	−0.042 (8)	−0.071 (5)	−0.014 (6)
F7A	0.122 (7)	0.117 (7)	0.116 (8)	−0.074 (6)	0.019 (6)	−0.071 (6)
F8A	0.105 (7)	0.063 (4)	0.131 (8)	−0.008 (4)	0.007 (5)	−0.043 (5)

### Geometric parameters (Å, °)

**Table:**

Cu1—O2	1.886 (3)	C13—C21	1.389 (7)
Cu1—O1	1.892 (3)	C13—H13	0.9300
Cu1—N2	2.008 (4)	C14—C15	1.336 (7)
Cu1—N1	2.013 (4)	C14—C21	1.425 (7)
Cu1—O5	2.320 (4)	C14—H14	0.9300
Cu2—O3	1.884 (3)	C15—C22	1.440 (7)
Cu2—O4	1.895 (3)	C15—H15	0.9300
Cu2—N4	1.990 (4)	C16—C17	1.350 (7)
Cu2—N3	2.004 (4)	C16—C22	1.407 (7)
Cu2—O6	2.363 (5)	C16—H16	0.9300
O1—C2	1.281 (5)	C17—C18	1.401 (7)
O2—C4	1.280 (5)	C17—H17	0.9300
O3—C7	1.269 (5)	C18—H18	0.9300
O4—C9	1.274 (5)	C19—C22	1.389 (6)
O5—H5D	0.842 (11)	C19—C20	1.419 (7)
O5—H5E	0.846 (11)	C20—C21	1.402 (6)
O6—H6D	0.852 (11)	C23—C24	1.380 (7)
O6—H6E	0.852 (11)	C23—H23	0.9300
O7—H7A	0.850 (11)	C24—C25	1.359 (8)
O7—H7B	0.845 (11)	C24—H24	0.9300
N1—C12	1.320 (6)	C25—C33	1.384 (8)
N1—C20	1.353 (6)	C25—H25	0.9300
N2—C18	1.316 (6)	C26—C27	1.327 (8)
N2—C19	1.372 (6)	C26—C33	1.445 (8)
N3—C23	1.325 (7)	C26—H26	0.9300
N3—C32	1.345 (6)	C27—C34	1.441 (8)
N4—C30	1.329 (6)	C27—H27	0.9300
N4—C31	1.348 (6)	C28—C34	1.381 (8)
C1—C2	1.501 (6)	C28—C29	1.380 (8)
C1—H1A	0.9600	C28—H28	0.9300
C1—H1B	0.9600	C29—C30	1.405 (7)
C1—H1C	0.9600	C29—H29	0.9300
C2—C3	1.399 (6)	C30—H30	0.9300
C3—C4	1.401 (7)	C31—C34	1.404 (7)
C3—C8	1.510 (6)	C31—C32	1.429 (7)
C4—C5	1.513 (6)	C32—C33	1.388 (7)
C5—H5A	0.9600	B1—F3	1.326 (6)
C5—H5B	0.9600	B1—F2	1.325 (6)
C5—H5C	0.9600	B1—F4A	1.328 (6)
C6—C7	1.499 (6)	B1—F1A	1.330 (6)
C6—H6A	0.9600	B1—F3A	1.343 (6)
C6—H6B	0.9600	B1—F1	1.356 (6)
C6—H6C	0.9600	B1—F4	1.363 (6)
C7—C8	1.392 (7)	B1—F2A	1.367 (6)
C8—C9	1.411 (6)	B2—F8	1.338 (5)
C9—C10	1.501 (7)	B2—F5A	1.340 (5)
C10—H10A	0.9600	B2—F8A	1.347 (6)
C10—H10B	0.9600	B2—F6	1.347 (5)
C10—H10C	0.9600	B2—F6A	1.349 (5)
C11—C13	1.356 (7)	B2—F7	1.351 (5)
C11—C12	1.394 (7)	B2—F5	1.357 (6)
C11—H11	0.9300	B2—F7A	1.358 (5)
C12—H12	0.9300		
			
O2—Cu1—O1	92.75 (14)	C11—C13—C21	120.1 (5)
O2—Cu1—N2	91.77 (16)	C11—C13—H13	119.9
O1—Cu1—N2	166.92 (15)	C21—C13—H13	119.9
O2—Cu1—N1	171.40 (16)	C15—C14—C21	121.8 (5)
O1—Cu1—N1	92.39 (16)	C15—C14—H14	119.1
N2—Cu1—N1	81.76 (17)	C21—C14—H14	119.1
O2—Cu1—O5	95.44 (15)	C14—C15—C22	121.1 (5)
O1—Cu1—O5	95.00 (14)	C14—C15—H15	119.5
N2—Cu1—O5	96.78 (16)	C22—C15—H15	119.5
N1—Cu1—O5	90.97 (16)	C17—C16—C22	119.2 (5)
O3—Cu2—O4	92.78 (14)	C17—C16—H16	120.4
O3—Cu2—N4	92.05 (17)	C22—C16—H16	120.4
O4—Cu2—N4	168.61 (17)	C16—C17—C18	120.3 (5)
O3—Cu2—N3	172.57 (17)	C16—C17—H17	119.9
O4—Cu2—N3	92.64 (17)	C18—C17—H17	119.9
N4—Cu2—N3	81.72 (19)	N2—C18—C17	122.4 (5)
O3—Cu2—O6	90.72 (16)	N2—C18—H18	118.8
O4—Cu2—O6	99.97 (17)	C17—C18—H18	118.8
N4—Cu2—O6	90.27 (18)	N2—C19—C22	123.3 (5)
N3—Cu2—O6	93.34 (17)	N2—C19—C20	115.9 (4)
C2—O1—Cu1	124.8 (3)	C22—C19—C20	120.9 (5)
C4—O2—Cu1	124.3 (3)	N1—C20—C21	123.1 (5)
C7—O3—Cu2	124.7 (3)	N1—C20—C19	117.0 (4)
C9—O4—Cu2	125.4 (3)	C21—C20—C19	119.9 (5)
Cu1—O5—H5D	108 (5)	C13—C21—C20	116.7 (5)
Cu1—O5—H5E	100 (4)	C13—C21—C14	125.0 (5)
H5D—O5—H5E	105 (6)	C20—C21—C14	118.3 (5)
Cu2—O6—H6D	109 (5)	C19—C22—C16	117.3 (5)
Cu2—O6—H6E	114 (5)	C19—C22—C15	118.1 (5)
H6D—O6—H6E	124 (7)	C16—C22—C15	124.7 (5)
H7A—O7—H7B	102 (8)	N3—C23—C24	122.2 (6)
C12—N1—C20	118.4 (4)	N3—C23—H23	118.9
C12—N1—Cu1	128.9 (4)	C24—C23—H23	118.9
C20—N1—Cu1	112.6 (3)	C25—C24—C23	119.7 (6)
C18—N2—C19	117.6 (5)	C25—C24—H24	120.2
C18—N2—Cu1	129.7 (4)	C23—C24—H24	120.2
C19—N2—Cu1	112.6 (3)	C24—C25—C33	120.0 (6)
C23—N3—C32	117.7 (5)	C24—C25—H25	120.0
C23—N3—Cu2	129.2 (4)	C33—C25—H25	120.0
C32—N3—Cu2	113.1 (4)	C27—C26—C33	122.6 (6)
C30—N4—C31	118.5 (5)	C27—C26—H26	118.7
C30—N4—Cu2	128.5 (4)	C33—C26—H26	118.7
C31—N4—Cu2	113.0 (4)	C26—C27—C34	121.1 (6)
C2—C1—H1A	109.5	C26—C27—H27	119.4
C2—C1—H1B	109.5	C34—C27—H27	119.4
H1A—C1—H1B	109.5	C34—C28—C29	119.2 (6)
C2—C1—H1C	109.5	C34—C28—H28	120.4
H1A—C1—H1C	109.5	C29—C28—H28	120.4
H1B—C1—H1C	109.5	C28—C29—C30	119.4 (6)
O1—C2—C3	126.3 (5)	C28—C29—H29	120.3
O1—C2—C1	113.0 (4)	C30—C29—H29	120.3
C3—C2—C1	120.7 (4)	N4—C30—C29	122.0 (6)
C2—C3—C4	120.8 (4)	N4—C30—H30	119.0
C2—C3—C8	121.0 (5)	C29—C30—H30	119.0
C4—C3—C8	118.2 (4)	N4—C31—C34	122.8 (6)
O2—C4—C3	126.2 (5)	N4—C31—C32	116.6 (5)
O2—C4—C5	112.3 (5)	C34—C31—C32	120.6 (5)
C3—C4—C5	121.4 (5)	N3—C32—C33	123.8 (6)
C4—C5—H5A	109.5	N3—C32—C31	115.6 (5)
C4—C5—H5B	109.5	C33—C32—C31	120.6 (6)
H5A—C5—H5B	109.5	C25—C33—C32	116.6 (6)
C4—C5—H5C	109.5	C25—C33—C26	126.0 (6)
H5A—C5—H5C	109.5	C32—C33—C26	117.4 (6)
H5B—C5—H5C	109.5	C28—C34—C31	118.2 (6)
C7—C6—H6A	109.5	C28—C34—C27	124.2 (6)
C7—C6—H6B	109.5	C31—C34—C27	117.7 (6)
H6A—C6—H6B	109.5	F1—B1—F4	105.4 (5)
C7—C6—H6C	109.5	F2—B1—F1	110.2 (5)
H6A—C6—H6C	109.5	F2—B1—F4	109.7 (6)
H6B—C6—H6C	109.5	F3—B1—F4	109.9 (6)
O3—C7—C8	125.9 (5)	F3—B1—F1	110.2 (6)
O3—C7—C6	113.3 (5)	F3—B1—F2	111.4 (6)
C8—C7—C6	120.8 (5)	F4A—B1—F1A	111.5 (5)
C7—C8—C9	121.6 (5)	F4A—B1—F3A	111.0 (6)
C7—C8—C3	119.1 (5)	F1A—B1—F3A	109.8 (6)
C9—C8—C3	119.3 (5)	F4A—B1—F2A	107.9 (6)
O4—C9—C8	125.1 (5)	F1A—B1—F2A	109.2 (6)
O4—C9—C10	113.5 (5)	F3A—B1—F2A	107.2 (5)
C8—C9—C10	121.4 (5)	F6—B2—F7	107.9 (5)
C9—C10—H10A	109.5	F6—B2—F5	108.2 (5)
C9—C10—H10B	109.5	F7—B2—F5	110.3 (7)
H10A—C10—H10B	109.5	F8—B2—F7	109.9 (5)
C9—C10—H10C	109.5	F8—B2—F6	112.0 (6)
H10A—C10—H10C	109.5	F8—B2—F5	108.6 (6)
H10B—C10—H10C	109.5	F5A—B2—F8A	109.6 (5)
C13—C11—C12	119.8 (5)	F5A—B2—F6A	109.5 (6)
C13—C11—H11	120.1	F8A—B2—F6A	110.4 (6)
C12—C11—H11	120.1	F5A—B2—F7A	111.7 (7)
N1—C12—C11	121.9 (5)	F8A—B2—F7A	107.8 (5)
N1—C12—H12	119.1	F6A—B2—F7A	107.8 (5)
C11—C12—H12	119.1		
			
O2—Cu1—O1—C2	19.5 (4)	C13—C11—C12—N1	−1.0 (9)
N2—Cu1—O1—C2	129.6 (7)	C12—C11—C13—C21	−0.8 (9)
N1—Cu1—O1—C2	−167.4 (4)	C21—C14—C15—C22	−0.1 (9)
O5—Cu1—O1—C2	−76.2 (4)	C22—C16—C17—C18	0.8 (9)
O1—Cu1—O2—C4	−23.7 (4)	C19—N2—C18—C17	0.0 (8)
N2—Cu1—O2—C4	168.6 (4)	Cu1—N2—C18—C17	−177.0 (4)
O5—Cu1—O2—C4	71.6 (4)	C16—C17—C18—N2	−0.3 (9)
O4—Cu2—O3—C7	−22.8 (4)	C18—N2—C19—C22	−0.2 (7)
N4—Cu2—O3—C7	167.5 (4)	Cu1—N2—C19—C22	177.3 (4)
O6—Cu2—O3—C7	77.2 (4)	C18—N2—C19—C20	179.0 (4)
O3—Cu2—O4—C9	16.7 (5)	Cu1—N2—C19—C20	−3.5 (5)
N4—Cu2—O4—C9	131.7 (8)	C12—N1—C20—C21	−1.0 (7)
N3—Cu2—O4—C9	−168.4 (5)	Cu1—N1—C20—C21	−177.3 (4)
O6—Cu2—O4—C9	−74.5 (5)	C12—N1—C20—C19	177.7 (5)
O1—Cu1—N1—C12	13.4 (5)	Cu1—N1—C20—C19	1.4 (6)
N2—Cu1—N1—C12	−178.3 (5)	N2—C19—C20—N1	1.4 (7)
O5—Cu1—N1—C12	−81.6 (5)	C22—C19—C20—N1	−179.3 (4)
O1—Cu1—N1—C20	−170.8 (3)	N2—C19—C20—C21	−179.9 (4)
N2—Cu1—N1—C20	−2.5 (3)	C22—C19—C20—C21	−0.6 (7)
O5—Cu1—N1—C20	94.2 (3)	C11—C13—C21—C20	1.7 (8)
O2—Cu1—N2—C18	−5.3 (5)	C11—C13—C21—C14	−176.9 (5)
O1—Cu1—N2—C18	−115.5 (7)	N1—C20—C21—C13	−0.8 (8)
N1—Cu1—N2—C18	−179.6 (5)	C19—C20—C21—C13	−179.4 (5)
O5—Cu1—N2—C18	90.4 (5)	N1—C20—C21—C14	177.9 (5)
O2—Cu1—N2—C19	177.5 (3)	C19—C20—C21—C14	−0.7 (7)
O1—Cu1—N2—C19	67.3 (8)	C15—C14—C21—C13	179.7 (5)
N1—Cu1—N2—C19	3.2 (3)	C15—C14—C21—C20	1.1 (8)
O5—Cu1—N2—C19	−86.8 (3)	N2—C19—C22—C16	0.8 (8)
O4—Cu2—N3—C23	9.3 (5)	C20—C19—C22—C16	−178.5 (5)
N4—Cu2—N3—C23	179.3 (6)	N2—C19—C22—C15	−179.2 (4)
O6—Cu2—N3—C23	−90.9 (5)	C20—C19—C22—C15	1.6 (7)
O4—Cu2—N3—C32	−171.6 (4)	C17—C16—C22—C19	−1.0 (8)
N4—Cu2—N3—C32	−1.5 (4)	C17—C16—C22—C15	178.9 (5)
O6—Cu2—N3—C32	88.3 (4)	C14—C15—C22—C19	−1.2 (8)
O3—Cu2—N4—C30	−5.9 (5)	C14—C15—C22—C16	178.8 (5)
O4—Cu2—N4—C30	−121.0 (9)	C32—N3—C23—C24	0.0 (9)
N3—Cu2—N4—C30	178.1 (5)	Cu2—N3—C23—C24	179.1 (5)
O6—Cu2—N4—C30	84.8 (5)	N3—C23—C24—C25	−0.9 (11)
O3—Cu2—N4—C31	177.7 (4)	C23—C24—C25—C33	0.3 (11)
O4—Cu2—N4—C31	62.6 (10)	C33—C26—C27—C34	3.0 (11)
N3—Cu2—N4—C31	1.7 (4)	C34—C28—C29—C30	−0.1 (11)
O6—Cu2—N4—C31	−91.6 (4)	C31—N4—C30—C29	0.5 (9)
Cu1—O1—C2—C3	−7.8 (7)	Cu2—N4—C30—C29	−175.7 (5)
Cu1—O1—C2—C1	172.9 (3)	C28—C29—C30—N4	0.0 (10)
O1—C2—C3—C4	−7.5 (8)	C30—N4—C31—C34	−1.0 (8)
C1—C2—C3—C4	171.7 (5)	Cu2—N4—C31—C34	175.7 (4)
O1—C2—C3—C8	172.2 (5)	C30—N4—C31—C32	−178.5 (5)
C1—C2—C3—C8	−8.6 (7)	Cu2—N4—C31—C32	−1.7 (6)
Cu1—O2—C4—C3	16.7 (7)	C23—N3—C32—C33	1.5 (9)
Cu1—O2—C4—C5	−166.0 (3)	Cu2—N3—C32—C33	−177.7 (5)
C2—C3—C4—O2	2.6 (8)	C23—N3—C32—C31	−179.7 (5)
C8—C3—C4—O2	−177.1 (5)	Cu2—N3—C32—C31	1.1 (6)
C2—C3—C4—C5	−174.4 (5)	N4—C31—C32—N3	0.4 (8)
C8—C3—C4—C5	5.9 (7)	C34—C31—C32—N3	−177.1 (5)
Cu2—O3—C7—C8	17.2 (8)	N4—C31—C32—C33	179.2 (5)
Cu2—O3—C7—C6	−163.6 (3)	C34—C31—C32—C33	1.8 (9)
O3—C7—C8—C9	2.2 (9)	C24—C25—C33—C32	1.1 (10)
C6—C7—C8—C9	−176.9 (5)	C24—C25—C33—C26	−179.9 (6)
O3—C7—C8—C3	−176.4 (5)	N3—C32—C33—C25	−2.1 (9)
C6—C7—C8—C3	4.5 (8)	C31—C32—C33—C25	179.1 (5)
C2—C3—C8—C7	−79.9 (6)	N3—C32—C33—C26	178.8 (5)
C4—C3—C8—C7	99.8 (6)	C31—C32—C33—C26	0.0 (9)
C2—C3—C8—C9	101.5 (6)	C27—C26—C33—C25	178.5 (7)
C4—C3—C8—C9	−78.8 (6)	C27—C26—C33—C32	−2.5 (10)
Cu2—O4—C9—C8	−4.4 (8)	C29—C28—C34—C31	−0.4 (10)
Cu2—O4—C9—C10	175.4 (3)	C29—C28—C34—C27	179.2 (6)
C7—C8—C9—O4	−9.1 (9)	N4—C31—C34—C28	1.0 (9)
C3—C8—C9—O4	169.5 (5)	C32—C31—C34—C28	178.3 (6)
C7—C8—C9—C10	171.0 (5)	N4—C31—C34—C27	−178.6 (5)
C3—C8—C9—C10	−10.4 (8)	C32—C31—C34—C27	−1.3 (8)
C20—N1—C12—C11	1.9 (8)	C26—C27—C34—C28	179.3 (7)
Cu1—N1—C12—C11	177.5 (4)	C26—C27—C34—C31	−1.1 (10)

### Hydrogen-bond geometry (Å, °)

**Table:**

*D*—H···*A*	*D*—H	H···*A*	*D*···*A*	*D*—H···*A*
O7—H7B···F1A^i^	0.85 (1)	1.91 (2)	2.737 (11)	166 (8)
O7—H7A···F6A	0.85 (1)	1.90 (3)	2.728 (9)	164 (8)
O7—H7A···F7	0.85 (1)	1.99 (4)	2.782 (8)	155 (8)
O6—H6E···F4^ii^	0.85 (1)	2.05 (2)	2.898 (9)	171 (7)
O6—H6D···F2A^iii^	0.85 (1)	2.08 (2)	2.930 (10)	175 (7)
O6—H6D···F2^iii^	0.85 (1)	1.90 (3)	2.694 (10)	155 (7)
O5—H5E···F3A	0.85 (1)	1.96 (2)	2.776 (8)	163 (6)
O5—H5D···O7^iv^	0.84 (1)	2.00 (2)	2.837 (7)	173 (6)
O7—H7A···F7	0.85 (1)	1.99 (4)	2.782 (8)	155 (8)
O7—H7A···F6A	0.85 (1)	1.90 (3)	2.728 (9)	164 (8)
C29—H29···Cg^ii^	0.93	2.75	3.522 (7)	141

Symmetry codes: (i) −*x*+1, −*y*+1, −*z*+1; (ii) −*x*+2, −*y*+1, −*z*+1; (iii) *x*, *y*+1, *z*; (iv) *x*+1, *y*, *z*.
